# Expression of podoplanin and vimentin is correlated with prognosis in esophageal squamous cell carcinoma

**DOI:** 10.3892/mmr.2015.3966

**Published:** 2015-06-19

**Authors:** MAKIKO TANAKA, HIROSHI KIJIMA, HIDEO SHIMADA, HIROYASU MAKUUCHI, SOJI OZAWA, SADAKI INOKUCHI

**Affiliations:** 1 Department of Critical Care and Emergency Medicine, Tokai University School of Medicine, Isehara, Kanagawa 259-1193, Japan; 2 Department of Pathology and Bioscience, Hirosaki University Graduate School of Medicine, Hirosaki, Aomori 036-8562, Japan; 3 Department of Gastroenterological Surgery, Tokai University School of Medicine, Isehara, Kanagawa 259-1193, Japan

**Keywords:** podoplanin, vimentin, prognosis, esophagus, squamous cell carcinoma

## Abstract

Podoplanin is a small membrane mucin, which is involved in cell migration and cancer cell invasion. However, the roles of podoplanin in esophageal squamous cell carcinoma (ESCC) are poorly understood. In the present study, 139 cases of surgically resected ESCC were analyzed and the clinicopathological significance of podoplanin membrane expression in ESCC was demonstrated. Podoplanin expression was positive in 66.2% (92/139) of ESCC samples; with weak expression in 32.4% (45/139), and strong expression in 33.8% (47/139). Membrane expression of podoplanin was significantly associated with tumor status (P=0.001), venous invasion (P=0.035) and Union for International Cancer Control stage (P=0.029). Patients who exhibited strong podoplanin expression, were shown to have a poorer prognosis [hazard ratio (HR), 3.949; 95% confidence interval (CI), 2.001–7.794]. Expression of vimentin, a mesenchymal marker, was detected in 49 cases (35.3%) and was associated with venous invasion (P=0.020). Vimentin-positive cases were also more likely to have a worse prognosis than vimentin-negative cases (HR, 2.161; 95% CI, 1.300–3.592). Podoplanin membrane expression was significantly correlated with vimentin cytoplasmic expression in ESCC (P<0.001). The present study confirmed that podo-planin and vimentin are independent predictors of mortality (HR, 3.084; 95% CI, 1.543–6.164). These results suggest that podoplanin membrane expression results in epithelial-mesenchymal transition and promotes aggressive invasion in human ESCC.

## Introduction

Esophageal squamous cell carcinoma (ESCC) is a common malignant tumor of the digestive tract and is associated with a poor prognosis (1,2). Patients with ESCC often have a poor outcome as it is a difficult disease to diagnose at an early stage (3,4). Advanced esophageal cancer carries a particularly unfavorable prognosis due to the rapid spread of the tumor beyond the esophageal wall (5,6). A treatment strategy for early ESCC has been established based around endoscopic procedures, and standard curative surgery is now performed in advanced ESCC. In Japan, the total 5-year survival rate of patients with ESCC is ~50%; that of patients with early ESCC is >70%, while in advanced cases it is ~20%, due to the high incidence of recurrence, metastasis and invasion of adjacent organs (3,7). The prognosis of ESCC remains poor despite recent therapeutic advances. Therefore, further clinicopathological studies are required in order to analyze the mechanisms underlying local invasion and metastasis in ESCC.

Podoplanin, a 38 kDa transmembrane protein, was identified as an independent platelet aggregation-inducing factor, and is known to be a specific marker for lymphatic vessels (8). A recent study reported that podoplanin promoted epithelial-mesenchymal transition (EMT) (9). It has also been shown to stimulate collective cell invasion and migration without EMT, by inducing a rearrangement of the actin cytoskeleton of MCF7 breast cancer cells (10). A number of studies have reported that podoplanin expression is correlated with lymph node metastasis, disease stage, lymphatic and vascular invasion, recurrence and a poor prognosis in ESCC (11,12). Vimentin is an important protein constituent of cellular intermediate filaments in normal and tumor mesenchymal cells (13,14). Therefore, vimentin expression is one of the primary indicators of the development of EMT in carcinomas, which suggests a tumor with an aggressive phenotype with invasive and/or metastatic potential (15). Vimentin expression in ESCC has also been shown to be an independent predictor of lymph node metastasis (16,17). However, to the best of our knowledge, there have been no clinicopathological studies investigating podoplanin and vimentin expression together in ESCC. The present study focused on the expression of podoplanin in the membrane, and its clinicopathological significance in the progression of ESCC.

## Materials and methods

### Patients

In total, 139 patients with ESCC who had undergone surgical resection of their tumor at Tokai University Hospital (Kanagawa, Japan) between January 2003 and December 2005, were enrolled into the study. The study was approved by the ethics committee of the Institutional Review Board of Tokai University Hospital (IRB no. 13R-33; Isehara, Japan). All participants provided informed consent, according to the Institutional Review Board of Tokai University Hospital. The 139 patients (128 males and 11 females; age range, 42–82 years; mean age, 63.4 years) with ESCC underwent surgery with three-field lymph node dissection and were not treated with radiotherapy or chemotherapy prior to surgery.

### Histopathological examination

Esophageal cancer specimens were routinely fixed with 10% formalin for 24–48 h, embedded in paraffin, cut into 4-*µ*m sections and stained with hematoxylin and eosin. The tumor stage was defined according to the tumor-node-metastasis classification of the Union for International Cancer Control (UICC) (18). In addition, histological factors were graded according to the Guidelines for Clinical and Pathological Studies on Carcinoma of the Esophagus (e.g. ly, v and INF). The degree of lymph node metastasis was classified as: n (−), metastasis negative; or n (+), metastasis positive. The degree of lymphatic invasion was classified as: ly0, no lymphatic invasion; ly1, mild lymphatic invasion; ly2, moderate lymphatic invasion; or ly3, severe lymphatic invasion. The degree of venous invasion was classified as: v (−), invasion negative; or v (+), invasion positive. Tumor infiltrative patterns (INF) at the invasive front were classified into three groups according to the general criteria described by the Japanese Gastric Cancer Association (19): INFa, cancer nests demonstrate expanding growth and have a clear border with pre-vesicular adipose tissue; INFb, cancer nests are intermediate between those of INFa and INFc; and INFc, scirrhous growth, in which cancer nests exhibit invasive growth and where the border with adipose tissue is unclear.

### Immunohistochemical analysis

An representative specimen for immunohistochemical analysis was selected from each patient with ESCC. Sections (5 *µ*m) were mounted on silane-coated glass slides, deparaffinized and autoclaved (ES-215, high-pressure steam sterilizer, TOMY, Tokyo, Japan) at 121°C for 10 min for antigen retrieval. The primary antibodies used in the immunohistochemical analyses were mouse monoclonal anti-podoplanin (clone D2–40; 3X dilution; cat. no. 413451; Nichirei Pharmaceutical, Tokyo, Japan) and mouse monoclonal anti-vimentin (clone v9; X100 dilution; cat. no. MO725; Dako Denmark A/S, Glostrup, Denmark). Immunoreactivity was detected using the avidin-biotin method (Vectastain Elite ABC kit, Vector Laboratories, Inc., Burlingame, CA, USA). Immunohistochemical images were captured using a digital microscope camera system (BX50 microscope and DP70 digital camera; Olympus Corporation, Tokyo, Japan).

### Evaluation of immunohistochemistry

**Figure 1:**
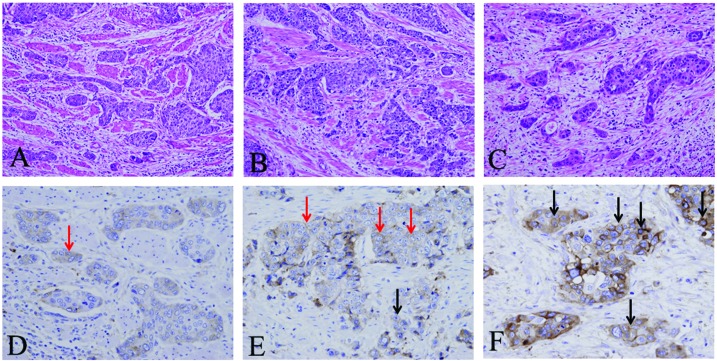
Immunohistochemical expression of podoplanin. Squamous cell carcinoma cells invasion of the stroma. Three representative cases, (A), (B) and (C). Hematoxylin and eosin staining (magnification, ×20). (D) Section from same sample as image (A). Negative, podoplanin is expression in incomplete membrane at ESCC. (E) Section from same sample as image (B). Weak, podoplanin is expression in incomplete membrane and/or complete membrane at ESCC. (F) Section from same sample as image (C). Strong, podoplanin is expression complete membrane at ESCC. Red arrows indicate expression in incomplete cell membrane, and black arrows are expression at complete cell membrane (magnification, ×40). ESCC, esophageal squamous cell carcinoma.

**Figure 2:**
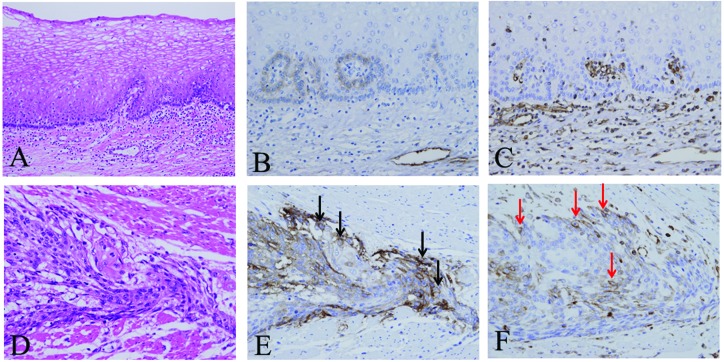
Immunohistochemical expression of podoplanin and vimentin. (A) Non-neoplastic esophageal mucosa consists of epithelium and lamina propria (H&E staining). (B) Podoplanin is strongly expressed in endothelial cells of lymphatic vessels but weakly expressed in the epithelial basal layers. (C) Vimentin staining was observed in the lamina propria, but was not detected in the epithelium. (D) Esophageal squamous cell carcinoma invasion of the stroma (H&E staining). (E) Podoplanin is strongly expressed at the invasive front of the carcinoma (black arrows). (F) Vimentin is expressed in a proportion of the carcinoma cells (red arrows), as well as in the stromal tissue (magnification, ×40). H&E, hematoxylin and eosin.

**Figure 3:**
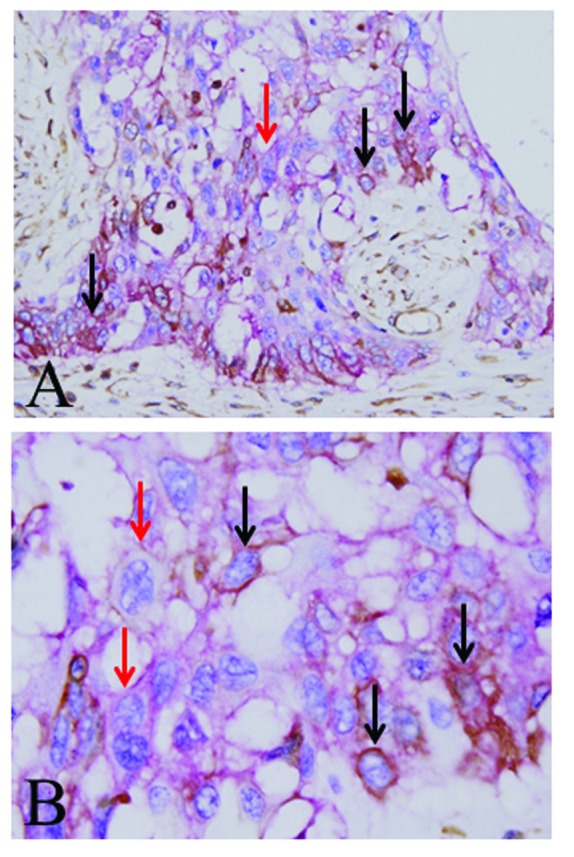
Double immunohistochemical staining of podoplanin (red phosphatase staining) and vimentin (brown peroxidase staining). A number of cancer cells expressed podoplanin and vimentin (black arrows). Podoplanin expression is observed at the cell membranes of carcinoma cells (red arrows). (A) Low power magnification (×40) and (B) high power magnification (×100).

For the analysis of the immunohistochemical expression of podoplanin, staining intensity was determined at the invasive front of ESCC (Fig. 1), and was classified into the following three criteria: 1) negative, incomplete membrane expression of <10% cancer cells at the invasive front (Fig. 1D); 2) weak, complete membrane expression of <10% cancer cells (Fig. 1E); 3) strong, complete membrane expression of ≥10% cancer cells (Fig. 1F). Vimentin expression was determined by the presence of cytoplasmic staining, in particular in the tumor/stoma interface at the invasive front of the cancer cells, and divided into the following two criteria: Negative, <10% vimentin expression at the invasive front of the cancer cells and positive, ≥10% vimentin expression at the invasive front (Fig. 1E).

### Statistical analysis

Univariate analysis was performed using the χ^2^ test. Cox proportional hazard regression analysis was used to determine the net effects of each predictor while controlling the effects of the other factors by uni- and multivariate analysis. Independent prognostic factors were analyzed by the Cox proportional hazard regression model. Hazard ratios (HRs) and associated 95% confidence intervals (CIs) were used to assess the independent contributions of significant factors. P<0.05 was considered to indicate a statistically significant difference. Overall survival was measured from the date of surgery to mortality from all causes. Survival curves were calculated using Kaplan-Meier methods and analyzed using the log-rank test. All analyses were performed using SPSS statistical software, version 21 (IBM SPSS, Armonk, NY, USA).

## Results

### Histological expression of podoplanin and vimentin

Podoplanin was strongly expressed in the endothelial cells of lymphatic vessels, which were examined as internal controls. Non-neoplastic mucosa, obtained from the surgically resected esophagus with ESCC, exhibited weak podoplanin expression at the basal layer adjacent to the connective tissue papillae. Podoplanin expression was positive in 66.2% (92/139) of samples from patients with ESCC (Fig. 2); weak expression was observed in 32.4% (45/139) and strong expression in 33.8% (47/139; Fig. 1). Vimentin expression was observed in the esophageal lamina propria, but was not detected in non-neoplastic esophageal epithelium (Fig. 2). Vimentin expression at the cancer invasive front was detected in 35.3% (49/139) of samples from patients with ESCC.

### Analysis of clinicopathological findings from ESCC using χ^2^ statistics

The associations between podoplanin and clinicopathological features, are summarized in Table I. Strong podoplanin expression was significantly correlated with tumor status (P=0.001), venous invasion (P=0.035) and UICC stage (P=0.029). The associations between Vimentin and clinicopathological features are summarized in Table II. Vimentin expression was significantly associated with venous invasion (P=0.020). The association between podoplanin and vimentin is summarized in Table III. Podoplanin membrane expression was strongly correlated with vimentin cytoplasmic expression in samples from patients with ESCC (P<0.001). Immunohistochemical double-staining demonstrated co-expression of podoplanin and vimentin in ESCC (Fig. 3).

### Correlation between prognosis and expression of podoplanin and vimentin in ESCC

**Figure 4:**
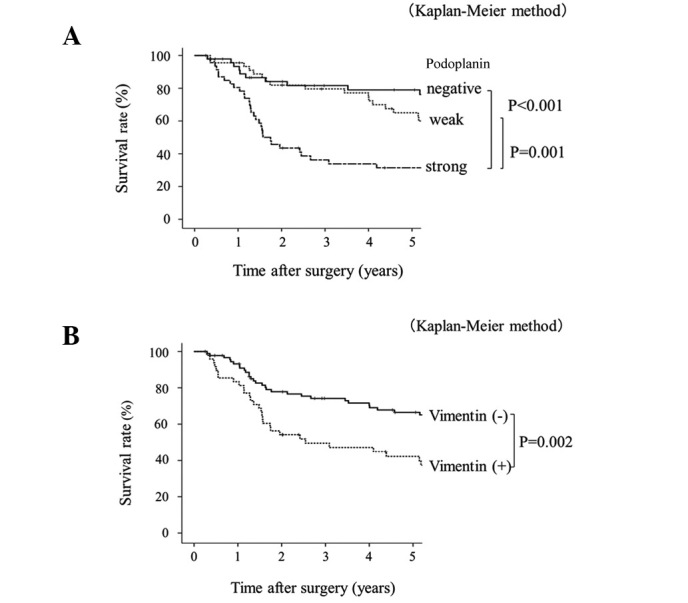
Kaplan-Meier survival curves. (A) Association between podoplanin expression and overall survival in ESCC. (B) Association between vimentin expression and survival in ESCC. ESCC, esophageal squamous cell carcinoma.

Patients exhibiting strong podoplanin expression had significantly poorer overall survival rates than those with negative or weak expression of podoplanin (<0.001, P=0.001, log-rank test, Fig. 4A). Patients from whom ESCC samples were vimentin-positive also had lower overall survival rates than patients who were negative for vimentin expression (P=0.002, log-rank test, Fig. 4B). Multivariate analysis demonstrated that strong podoplanin expression (HR, 3.084; 95% CI, 1.543–6.164) and lymph node metastasis (HR, 6.132; 95% CI, 2.355–15.916) were independent predictors of mortality in ESCC (Table IV). In addition, multivariate analysis showed that vimentin expression (HR, 2.008; 95% CI, 1.191–3.384), tumor status (HR, 1.830; 95% CI, 1.049–3.194) and lymph node metastasis (HR, 5.77; 95% CI, 2.221–15.028) were independent predictors of mortality in ESCC (Table V).

## Discussion

Recently, podoplanin has been shown to be a candidate marker for cancer stem cells, which is associated with cancer cell invasion and migration, as well as prognosis, in a number of types of cancer, including esophageal squamous cell carcinoma, lung squamous cell carcinoma and oral cancer (11,12,20–22). In the present study, the focus was on the expression of podoplanin in the cell membrane at the invasive front of ESCC tumors, since the podoplanin molecule is a transmembrane protein. The expression of podoplanin on the cell membrane was positively correlated with tumor status, venous invasion and UICC stage, and was associated with a poor prognosis in ESCC. Strong podoplanin membrane expression was an independent predictor of mortality in patients with ESCC (HR 3.084, 95% CI, 1.543–6.164). To the best of our knowledge, this is the first study to report the association between podoplanin membrane expression and the degree or presence of certain clinicopathological factors. Studies have previously described podoplanin expression in cancer cells, but have not discussed podoplanin membrane expression specifically (11,12).

Rahadiani *et al* (11) reported that high podoplanin expression was significantly correlated with tumor status, depth of invasion, and lymphatic and vascular invasion, and was associated with a poorer prognosis in ESCC. These authors demonstrated that podoplanin was involved in cancer cell invasion and tumorigenesis through the use of experimental procedures, such as the Matrigel invasion assay and an *in vivo* mouse study. Tong *et al* (12) reported that podoplanin expression was correlated with lymph node metastasis, UICC stage and the immunoreactivity score, and was associated with a poor prognosis in ESCC.

Several studies have clarified that podoplanin expression promotes EMT at molecular/histopathological levels (9). The present study focused on membrane expression of podoplanin, as membrane expression has been suggested to have significant roles in EMT (9) and cell invasion processes (10). In cancer cells, EMT is a phenotypic change, by which epithelial cells lose their polarity and epithelial markers, such as E-cadherin, and acquire migratory factors that are characteristic of fibroblasts, such as snail and vimentin (23–27). Vimentin expression is an important indicator of EMT in carcinomas (27). This transition suggests the development of an aggressive phenotype with increased invasive and metastatic potential (17). Increased vimentin expression has been reported in a number of epithelial cancers, including breast cancer, lung cancer and esophageal squamous cell carcinoma (16,28,29). In the present study, it was also demonstrated that vimentin expression is associated with venous invasion and that it is an independent predictor of prognosis in ESCC. It is hypothesized that podoplanin is likely to be important in the process of EMT, as a number of cancer cells in ESCC co-expressed podoplanin and vimentin. A proportion of podoplanin-positive cancer cells also expressed vimentin, suggesting that podoplanin may result in vimentin-associated EMT. EMT is understood to induce a more aggressively invasive and malignant phenotype in ESCC. Future studies should also analyze the expression of EMT markers, such as N-cadherin, snail and fibronectin, and the epithelial marker, E-cadherin, in ESCC. Finally, in the present study, the membrane expression of podoplanin was shown to be a novel immunohistochemical indicator of a highly malignant phenotype of human ESCC.

## Figures and Tables

**Table I:** Podoplanin membrane expression and clinicopathological factors of esophageal squamous cell carcinoma.

Variable	Number of patients	Podoplanin staining intensity			P-value
		Negative	Weak	Strong	
Age at surgery (years)					0.406
<63	66 (47.5)	21 (31.8)	19 (28.8)	26 (39.4)	
≥63	73 (52.5)	26 (35.6)	26 (35.6)	21 (28.8)	
Gender					0.696
Male	128 (92.1)	42 (32.8)	42 (32.8)	44 (34.4)	
Female	11 (7.9)	5 (45.4)	3 (27.3)	3 (27.3)	
Tumor status					0.001
T1	48 (34.5)	24 (50.0)	17 (35.4)	7 (14.6)	
T2	25 (18.0)	4 (16.0)	13 (52.0)	8 (32.0)	
T3	61 (43.9)	19 (31.1)	14 (23.0)	28 (45.9)	
T4	5 (3.6)	0 (0.0)	1 (20.0)	4 (80.0)	
Lymph node metastasis					0.164
n (−)	43 (30.9)	18 (40.9)	16 (36.4)	10 (22.7)	
n (+)	96 (69.1)	29 (30.5)	29 (30.5)	37 (39.0)	
Lymphatic invasion					0.870
ly (0, 1)	87 (62.6)	30 (34.5)	29 (33.3)	28 (32.2)	
ly (2, 3)	52 (37.4)	17 (32.7)	16 (30.8)	19 (36.5)	
Venous invasion					0.035
v (−)	52 (37.4)	23 (44.2)	18 (34.6)	11 (21.2)	
v (+)	87 (62.6)	24 (27.6)	27 (31.0)	36 (41.4)	
Histological differentiation					0.159
Well	38 (27.3)	9 (23.7)	13 (34.2)	16 (42.1)	
Mod	77 (55.4)	25 (32.4)	26 (33.8)	26 (33.8)	
Poor	24 (17.3)	13 (54.2)	6 (25.0)	5 (20.8)	
Infiltration pattern					0.398
inf a, b	97 (69.8)	36 (37.1)	31 (32.0)	30 (30.9)	
inf c	42 (30.2)	11 (26.2)	14 (33.3)	17 (40.5)	
UICC stage					0.029
IA, IB	37 (26.6)	16 (43.2)	14 (37.9)	7 (18.9)	
IIA, IIB	36 (25.9)	10 (27.8)	16 (44.4)	10 (27.8)	
IIIA, IIIB, IIIC	66 (47.5)	21 (31.8)	15 (22.7)	30 (45.5)	

Numbers in brackets represent the percentages of patients/samples in that category. n, lymph node metastasis; ly, lymphatic invasion; v, venous invasion; UICC, Union for International Cancer Control.

**Table II:** Vimentin expression and clinicopathological factors of esophageal squamous cell carcinoma.

Variable	Number of patients	Vimentin (−)	Vimentin (+)	P-value
Age at surgery (years)				0.794
<63	66 (47.5)	42 (63.6)	24 (36.4)	
≥63	73 (52.5)	48 (65.8)	25 (34.2)	
Gender				0.163
Male	128 (92.1)	85 (66.4)	43 (33.6)	
Female	11 (7.9)	5 (45.5)	6 (54.5)	
Tumor status				0.143
T1	48 (34.5)	37 (77.1)	11 (22.9)	
T2	25 (18.0)	16 (64.0)	9 (36.0)	
T3	61 (43.9)	34 (55.7)	27 (44.3)	
T4	5 (3.6)	3 (60.0)	2 (40.0)	
Lymph node metastasis				0.180
n (−)	44 (31.7)	32 (72.7)	12 (27.3)	
n (+)	95 (68.3)	58 (61.1)	37 (38.9)	
Lymphatic invasion				0.178
ly (0, 1)	87 (62.6)	60 (69.0)	27 (31.0)	
ly (2, 3)	52 (37.4)	30 (57.7)	22 (42.3)	
Venous invasion				0.020
v (−)	52 (37.4)	40 (76.9)	12 (23.1)	
v (+)	87 (62.6)	50 (57.5)	37 (42.5)	
Histological differentiation				0.582
Well	38 (27.3)	22 (57.9)	16 (42.1)	
Mod	77 (55.4)	52 (67.5)	25 (32.5)	
Poor	24 (17.3)	16 (66.7)	8 (33.3)	
Infiltration pattern				0.105
inf a, b	97 (69.8)	67 (69.1)	30 (30.9)	
inf c	42 (30.2)	23 (54.8)	19 (45.2)	
UICC stage				0.231
IA, IB	37 (26.6)	27 (73.0)	10 (27.0)	
IIA, IIB	36 (25.9)	25 (69.4)	11 (30.6)	
IIIA, IIIB, IIIC	66 (47.5)	38 (57.6)	28 (42.4)	

Numbers in brackets represent the percentages of patients/samples in that category. n, lymph node metastasis; ly, lymphatic invasion; v, venous invasion; UICC, Union for International Cancer Control.

**Table III:** Podoplanin membrane expression and vimentin expression in esophageal squamous carcinoma.

Variable	Number of patients	Podoplanin staining intensity			P-value
		Negative	Weak	Strong	
Vimentin					<0.001
Negative	90 (64.7)	42 (46.7)	31 (34.4)	17 (18.9)	
Positive	49 (35.3)	5 (10.2)	14 (28.6)	30 (61.2)	

Numbers in brackets represent the percentages of patients/samples in that category.

**Table IV:** Multivariate analysis of clinicopathological factors and patients' survival of esophageal squamous cell carcinoma.

Variable	Number of patients	Hazard ratio	95% confidence interval	P-value
Podoplanin staining intensity				
Negative (reference)	47 (33.8)			
Weak	45 (32.4)	1.509	0.717–3.174	0.278
Strong	47 (33.8)	3.084	1.543–6.164	0.001
Age at surgery (years)				0.551
<63	66 (47.5)	0.856	0.514–1.426	
≥63	73 (52.5)			
Gender				0.604
Male	128 (92.1)	0.685	0.164–2.868	
Female	11 (7.9)			
Tumor status				0.123
T1, T2	73 (52.5)	1.587	0.882–2.854	
T3, T4	66 (47.5)			
Lymph node metastasis				<0.001
n (−)	44 (31.7)	6.123	2.355–15.916	
n (+)	95 (68.3)			

Numbers in brackets represent the percentages of patients/samples in that category. n, lymph node metastasis.

**Table V:** Multivariate analysis of clinicopathological factors and patients' survival of esophageal squamous cell carcinoma.

Variable	Number of patients	Hazard ratio	95% confidence interval	P-value
Vimentin				0.009
Negative	90 (64.7)	2.008	1.191–3.384	
Positive	49 (35.3)			
Age at surgery (years)				0.528
<63	66 (47.5)	0.849	0.510–1.412	
≥63	73 (52.5)			
Gender				0.266
Male	128 (92.1)	0.441	0.104–1.865	
Female	11 (7.9)			
Tumor status				0.033
T1, T2	73 (52.5)	1.830	1.049–3.194	
T3, T4	66 (47.5)			
Lymph node metastasis				<0.001
n (−)	90 (64.7)	5.777	2.221–15.028	
n (+)	49 (35.3)			

Numbers in brackets represent the percentages of patients/samples in that category. n, lymph node metastasis.
